# A novel tumor suppressor function of Kindlin-3 in solid cancer

**DOI:** 10.18632/oncotarget.2125

**Published:** 2014-06-18

**Authors:** Ibtissem Djaafri, Farah Khayati, Suzanne Menashi, Jorg Tost, Marie-Pierre Podgorniak, Aurelie Sadoux, Antoine Daunay, Luis Teixeira, Jean Soulier, Ahmed Idbaih, Niclas Setterblad, Françoise Fauvel, Fabien Calvo, Anne Janin, Celeste Lebbé, Samia Mourah

**Affiliations:** ^1^ Inserm UMR-S 940 Paris, F-75010, France; ^2^ Institute of Hematology (IUH), Université Paris-Diderot, Sorbonne Paris Cité, Paris, F-75010, France; ^3^ AP-HP, Hôpital Saint-Louis, Laboratoire de Pharmacologie-Génétique, Paris, F-75010, France; ^4^ Université Paris Est Créteil, CNRS-UMR 7149, Créteil, France; ^5^ Laboratory for Epigenetics, Centre National de Génotypage, CEA-Institut de Génomique, Evry, France; ^6^ Laboratory for Functional Genomics, Fondation Jean Dausset - CEPH, Paris, F-75010, France; ^7^ AP-HP; Hôpital Saint-Louis, Service d'oncologie médicale, Paris, F-75010, France; ^8^ Hematology Laboratory APHP, Saint-Louis Hospital, Paris F-75010, France; ^9^ AP-HP, Groupe Hospitalier Pitié-Salpêtrière, Service de Neurologie 2-Mazarin, Paris, France; ^10^ Inserm U 975, Paris, 75013 France, CNRS, UMR 7225, Paris, France; ^11^ Inserm, U728, Paris, F-75010, France; ^12^ AP-HP, Hôpital Saint-Louis, Laboratoire de Pathologie, Paris, F-75010, France; ^13^ AP-HP, Hôpital Saint-Louis, Département de Dermatologie, Paris, F-75010, France; ^14^ Inserm U976, Paris, F-75010, France

**Keywords:** Tumor suppressor gene, Kindlin-3, Invasion/Migration, metastasis, Integrins

## Abstract

Kindlin-3 (FERMT-3) is known to be central in hemostasis and thrombosis control and its deficiency disrupts platelet aggregation and causes Leukocyte Adhesion Deficiency disease. Here we report that Kindlin-3 has a tumor suppressive role in solid cancer. Our present genetic and functional data show that Kindlin-3 is downregulated in several solid tumors by a mechanism involving gene hypermethylation and deletions. *In vivo* experiments demonstrated that Kindlin-3 knockdown in 2 tumor cell models (breast cancer and melanoma) markedly increases metastasis formation, in accord with the in vitro increase of tumor cell malignant properties. The metastatic phenotype was supported by a mechanism involving alteration in β3-integrin activation including decreased phosphorylation, interaction with talin and the internalization of its active form leading to less cell attachment and more migration/invasion. These data uncover a novel and unexpected tumor suppressor role of Kindin-3 which can influence integrins targeted therapies development.

## INTRODUCTION

Kindlin-3 (Mig2B, FERMT-3), a key integrin activating protein belongs to the Kindlin family which includes three members that share considerable sequence and structural homologies. They are encoded by three different genes, *KIND1* (*FERMT1*, chromosome 20p12.3), *KIND2* (*FERMT2*, chromosome 14q22.1), *KIND3* (*FERMT3*, chromosome 11q13.1). Kindlins are characterized by a C-terminal domain FERM (4.1 protein, ezrin, radixin, moesin) which is bisected by a pleckstrin homology domain [1-4]. Kindlins can bind directly to various classes of integrins and participate in their activation, thus playing a key role in the regulation of cell-matrix junctions, such as focal adhesions, as well as cell-cell contacts. Kindlin-3 has been mainly described in hematopoietic cells and appears to be central in the control of hemostasis and thrombosis [5, 6]. It has been implicated in β2 integrin activation in leukocytes and was shown to promote their adhesion and endothelial transmigration [5]. Point mutations in the *kindlin-3* gene have been identified in humans with a rare inherited Integrin Activation Deficiency Disease (IADD), also designated as Leukocyte Adhesion Deficiency syndrome (LAD-III or LADI variant). The manifestations of the Kindlin-3 deficiency include episodic bleeding, susceptibility to frequent infections and osteopetrosis which result from an inability to activate β1, β2 and β3 integrins [5, 7-10]. A recent study reported that Kindlin-3 is also expressed in endothelial cells and contributes to their integrin-mediated adhesion [11]. Kindlin-3 knockdown in endothelial cells results in impaired adhesion to integrin substrates, despite the presence of Kindlin 2 in these cells suggesting different roles for these Kindlin members in integrin signaling [11].

While Kindlin-3 expression is restricted to cells of hematopoietic origin, Kindlin-1 is predominantly expressed in epithelial cells in tissues such as skin, intestine and kidney and Kindkin-2 is expressed in most tissues but mainly in skeletal and smooth muscle cells [4]. All three proteins are localized to integrin-dependent adhesion sites.

Integrins play key roles in cell adhesion by providing a physical connection between the extracellular matrix and the cytoskeleton. They have also been shown to regulate intracellular signaling processes involved in migration, invasion, proliferation, differentiation, and survival of normal and tumor cells [12-14]. In particular, integrin β3 has been suggested to have prominent functions in cancer biology, its expression in tumor tissue correlates with tumor progression and it was shown to contribute to the metastasis of different cancer types [15-18]. Integrins exist in two main conformational states, upon ligand binding and cellular stimulation, integrins are activated by shifting from a low affinity to a high affinity state and this was shown to require the binding of several adaptor proteins to the cytoplasmic domain of integrin. It is now widely accepted that both of the cytoplasmic proteins kindlins and talins are critical for integrin activation through binding to the cytoplasmic tail of the integrin subunit [4].

Talin-mediated integrin activation was suggested to play a role in tumor progression and talin expression was shown to increase significantly in certain cancers and to correlate with progression to the metastatic disease [19]. Both kindlin-1 and kindlin-2, apart from their implication in inherited disease, have also been reported to be deregulated in certain human cancers [20]. However, the role of Kindlin-3 in solid tumors has not yet been explored, probably because it was thought to be only expressed by hematopoietic cells. Since our initial studies detected Kindlin-3 expression in several cell and tissue types, albeit in lower levels than in platelets and leukocytes, and in view of its ability to crosstalk with integrins, having crucial role in tumorigenesis [21] we hypothesized that Kindlin-3 could play a role in cancer.

## RESULTS

### Kindlin-3 is down-expressed in human tumors

**Figure 1:**
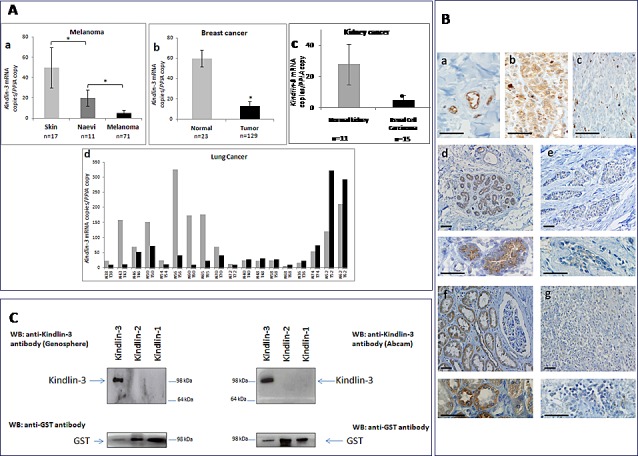
Kindlin-3 expression in human tumors **A. Downregulation in Kindlin-3 mRNA observed in human tumors from several origins compared to normal corresponding tissues** Relative expression of *Kindlin-3* mRNA to the reference gene *PPIA* assessed by qRT-PCR (a) Melanoma tissues compared to benign melanocytic naevi and normal skin tissues (n=71, n=11 and n= 17 respectively); (b) Primary breast tumors compared to normal breast tissues (n=129 and n= 23 respectively); (c) Kidney cancers compared to normal kidney (n=15 and n= 11 respectively); (d) 18 matched pairs of lung cancer versus normal lung tissues from the same patients. *P* values were calculated using two-sided Student *t* test. Means and 95% confidence intervals are shown;*, *P* <0.05. **B. Immunohistochemical staining of Kindlin-3 in sections of human cancer and normal tissues** (a) Expression of *Kindlin-3* in endothelial cells within blood vessels, as positive control; (b and c) naevi and melanoma; (d and e) normal and breast cancer (f and g) normal and kidney cancer respectively. Higher magnification images are shown below each image for breast and kidney tissues. The illustrated immunostaining shown was performed with the Genosphere anti-Kindlin-3 Ab. Images are representative of 6 tumor specimens for each cancer type. Similar staining was obtained using the Abcam anti-Kindlin-3 Ab. Note that staining is always lower in the corresponding cancer tissues. Scale bar = 20μm. **C. Specificity of the anti-Kindlin-3 antibodies used** Western blot analyses of N-terminal GST-tagged recombinant Kindlin-1 (300ng), Kindlin-2 (300ng) or Kindlin-3 (150ng) proteins (H00055612-P01, H00010979-P01, H00083706-P01; Novus Biologicals Cambridge, UK). Blots were probed with 2 different kindlin-3 antibodies (upper panels) or GST antibody; Millipore) (lower panels). A single band corresponding to GST-tagged Kindlin-3 was obtained with both antibodies used, ab68040 (Abcam, Cambridge MA, USA) and the custom made anti-Kindlin-3 antibody produced against a human Kindlin-3 specific peptide corresponding to amino acids 19 –31 (RVFVGEEDPEAES) and affinity purified on a sepharose matrix (Genosphere, France). Importantly, neither of the two antibodies recognized GST tagged Kindlin-1 or Kindlin-2 even when 2x quantity of these recombinant proteins was loaded.

We analyzed the expression of Kindlin-3 mRNA and protein in several tumor types including melanoma, breast, lung and kidney cancers and observed that Kindlin-3 was significantly downregulated in these tumors when compared with the normal tissue counterparts (Figure 1A). Histological examination of the tumor samples confirmed the predominance of malignant cells (> 80%), suggesting that the Kindlin-3 mRNA expression is mostly from tumor cell origin. Kindlin-3 transcript levels in breast cancer (n=129) were 5 fold lower than in normal breast tissues (n=23) (*P*< .001, Student *t* test). A similar profile was observed in a series of kidney cancer tissues (n=15 cancer vs 11 normals; *P*< .05). Kindlin-3 levels in nevi (n=11), benign cutaneous melanocytic proliferations which can sometimes progress to melanoma, was lower than in normal skin (n=17) but significantly higher than in melanoma lesions (n=71) (*P*< .05). Ten out of 18 matched pairs of lung cancer and normal tissues, derived from the same patients at a distance from the tumor, had Kindlin-3 transcriptionally down-regulated in the cancer tissues. These expression data were confirmed by the histological analysis using specific anti-Kindlin-3 antibodies which do not recognize Kindlin-1 or Kindlin-2 (Figure 1B-C). Furthermore a significant correlation was observed between Kindlin-3 transcript and protein expression when examined in a series of 18 tumors (Spearman r = 0.62 P<0.05). Taken together, these results suggest that Kindlin-3 downregulation may be a common phenomenon in solid cancers. This assertion is further supported by the decreased expression of Kindlin-3 described in the Oncomine microarray database for lung, colon cancers and melanoma totaling 424 individual cases (Supplementary Figure 1).

### Kindlin-3 gene regulation in human tumors

**Figure 2:**
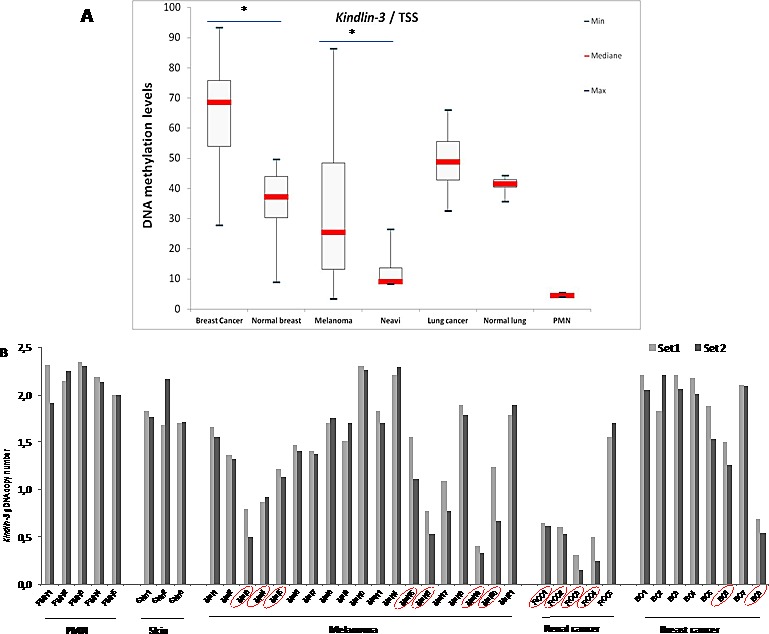
Kindlin-3 gene regulation in human tumors A **Kindlin-3 DNA methylation as a candidate mechanism for inactivation in cancer: DNA methylation levels in cancer versus normal tissues** Boxplots obtained by the analysis of the median levels at the TSS (transcription start site) (AMP2). DNA methylation levels in tumor tissues are compared to normal corresponding samples as well as to peripheral mononuclear cells (PMN). Median levels are shown by the bar, boxplots delineate the Interquartile range; bars show the minimal and maximal values. Differences were assessed by the non-parametric Mann-Whitney Test; *, *P* <0.05. **B. Kindlin-3 deletion identified as a candidate mechanism for Kindlin-3 inactivation in cancer** Copy-number variations (CNVs) of gDNAs using qPCR (grey and black by *Kindlin-3* primer set 1 and 2, respectively) and recurrence across distinct melanoma, kidney and breast cancer patients (positives surrounded or circled in red). PMN and skin samples were used for diploid gDNAs.

Promoter methylation is one of the pivotal mechanisms which contribute to gene silencing in tumor progression. The methylation status of three regions of potential interest in the *kindlin-3* gene, including two CpG islands as well as the TSS (transcription start site), was investigated by serial pyrosequencing on bisulfite-treated DNA obtained from breast, melanoma and lung cancer tissues and the corresponding non-neoplastic tissues. *Kindlin-3* promoter was significantly hypermethylated at the TSS (Fig. 2A) as well as in the first intron (supplementary Figure 2a) in most cancer samples analysed (breast cancer vs normal breast *P*=0.0006 and 0.02; melanoma vs nevi *P*=0.0055 and 0.03 respectively). High methylation levels of *Kindlin-3* in these regions were also observed in most cultured tumor cells (melanoma, breast and lung cell lines) (Supplementary Figure 2b).

We next examined whether deletions in *Kindlin-3* gene may also contribute to its silencing. Preliminary screening for potential deletions in *kindlin-3* gene (11q13.1 loci), using Agilent CGH 180K array containing two Kindlin-3 specific probes revealed a genomic loss in 2/8 melanoma tissues. Similar frequency of focal deletion was obtained for the tumor suppressor *CDKN2A* (p16). This analysis was then extended to a wider series of tumors using a more detailed mapping approach with genomic DNA (gDNA) quantification PCR (qPCR). Tumor specific *Kindlin-3* gene deletion was found in 7/19 melanoma; 2/8 breast cancer and 4/5 renal cell carcinoma analysed by copy number loss, relative to normal peripheral mononuclear cells (PMN) for diploid gDNA, producing either 1 or 2 fold decrease (corresponding to heterozygous or homozygous deletions) (Figure 2B). These findings were strengthened by The Cancer Genome Atlas dataset (TCGA) showing, in various subtypes of cancer, up to 12% of cases with homozygous deletion of chromosome region 11q13.1, including *Kindlin-3* gene (Supplementary Table-1) [22-25]. Interestingly, the CGH data retrieved from the Progenetix database showed a 33% recurring deletions this region in a cohort of 83 primary melanomas [26, 27].

**Figure 3:**
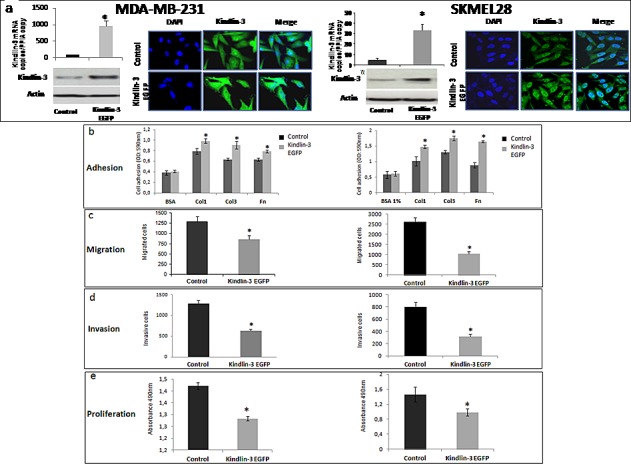
Kindlin-3 upregulation increases adhesion and decreases migration, invasion and proliferation in tumor cells a Breast cancer (MDA-MB-231) and melanoma (SKMEL-28) cell lines were transfected with pcDNA3-EGFP-Kindlin-3 (*Kindlin-3 EGFP*) or mock-vector (*Control*). Overexpression was confirmed by qRT-PCR (graphs showing means of relative expression to the reference gene *PPIA* of at least 3 independent experiments, error bars refer to 95% confidence intervals; *, *P* <0.05); and Immunofluorescence (IF). Representative IF images of three independent experiments are shown. b. Adhesion assay was performed with Kindlin*-3 EGFP* and *control* transfected cells seeded on fibronectin (Fn), collagen I (Col1) or collagen III (Col3). Adherent cells were quantified by staining with crystal violet which was solubilized with Sorenson solution, followed by OD reading at 540 nm. Bars represent means from four independent experiments carried out in triplicate; error bars refer to 95% confidence intervals; *, *P* <0.05. c and d. Cell migration and invasion were analysed by seeding Kindlin*-3 EGFP* and mock transfected MDA-MB-231 or SKMEL-28 cells in 12-well/insert plates (on uncoated filters for migration and on coated filters with matrigel for invasion). After 24h of incubation, cells were fixed, stained with Diff Quik and counted under a microscope. Columns, means of three independent experiments carried out in triplicate; error bars refer to 95% confidence intervals; *, *P* <0.05. e. Tumor cell proliferation was measured by bioreduction of tetrazolium (MTS). Results are presented as the mean of three independent experiments carried out in triplicate; error bars refer to 95% confidence intervals; *, *P* <0.05.

### Kindlin-3 regulates the malignant properties of tumor cells

**Figure 4:**
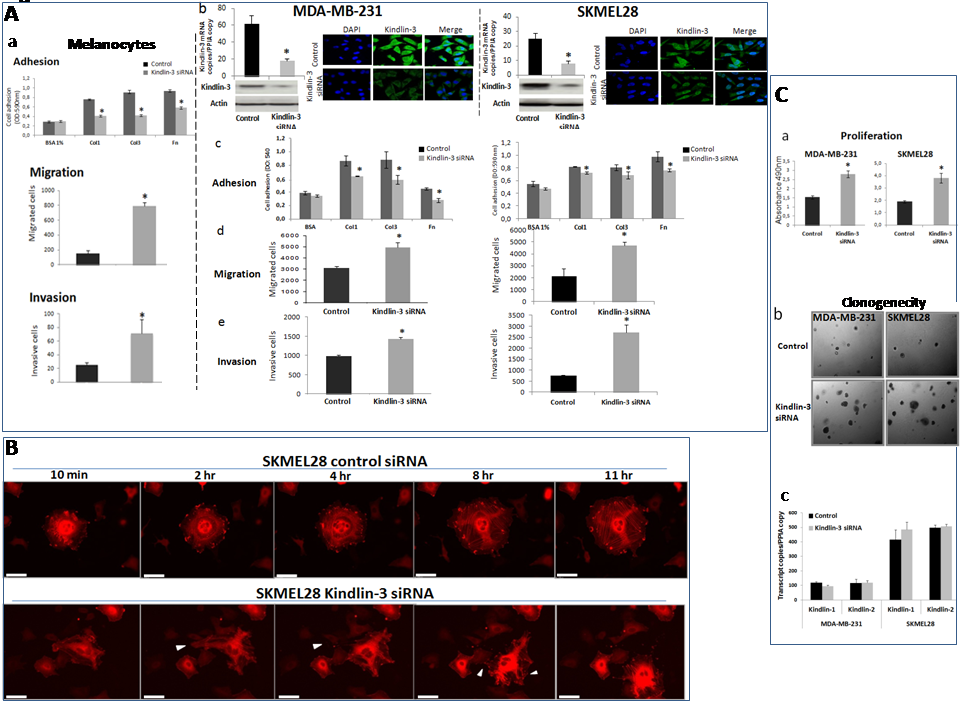
Kindlin-3 silencing enhances malignant properties of tumor cells A **Kindlin-3 silencing decreases cell adhesion and increases cell migration and invasion** a. **Melan-a (normal melanocytes) were silenced for** Kindlin*-3* (Kindlin*-3* siRNA) or transfected with scrambled siRNA (control) and subjected to cell adhesion assay on fibronectin (Fn), collagen I (Col1) or collagen III (Col3), to cell migration and to invasion assays. Bars represent means from three independent experiments carried out in triplicate; error bars refer to 95% confidence intervals; *, *P* <0.05. b-e. Tumor cells, breast (MDA-MB-231) and melanoma (SKMEL-28) were silenced by siRNA transfection for Kindlin*-3* expression. Kindlin-3 expression was analyzed by qRT-PCR and Immunofluorescence. They were then analysed for (c) adhesion, (d) migration and (e) invasion. Bars represent means from three independent experiments carried out in triplicate; error bars refer to 95% confidence intervals; *, *P* <0.05. **B. Time-lapse pictures of representative adhering/migrating live cells** SKMEL28 cells (Kindlin-3 siRNA and control siRNA transfected) transduced by CellLight® Actin-RFP were tracked for 24h (3min interval). Imaging was performed using 40x objective on a Nikon BioStation IM Live Cell Recorder. Time-lapse montages show representative images over a period of 11hr. Scale bar = 50 μm. Migrating cells were more often seen in Kindlin-3 siRNA cells with densely packed actin stress fibers and induction of filopodia formation in the direction of migration (arrows). Note that the filopodia seen on the left side of the cell at 2hr is no longer seen at 4hr (arrows), and that the cell moved to the right of the field at 11hr. Also, stress fibers appear disorganized, in particular at the later time points. By contrast, in control, more cells were engaged in adhesion process displaying a normal distribution of filamentous actin (actin in red). See corresponding supplemental movies 1 (control siRNA) and 2 (Kindlin-3 siRNA). **C. Kindlin-3 silencing enhances tumor cell growth and clonogenicity of tumor cells** a. The proliferation of MDA-MB-231 and SKMEL28 Kindlin-3 or control siRNA transfected cells was measured by MTS assay. Results are presented as the mean of three independent experiments carried out in triplicate; bars refer to 95% confidence intervals; *, *P* <0.05. b. Kindlin-3 siRNA increased the number of colonies formed with MDA-MB-231 and SKMEL-28 cells. Representative images of three independent experiments carried out in triplicate are shown. c. Kindlin-1 and Kindlin-2 expression analyzed by qRT-PCR using specific primer and probe sets were not altered by Kindlin-3 siRNA transfection. Columns, means of three independent experiments carried out in triplicate; bars refer to 95% confidence intervals.

The down regulation of Kindlin-3 in malignant tumors suggested that Kindlin-3 plays a role in controlling the malignant phenotype. To test this possibility, we modulated Kindlin-3 expression in non-malignant (normal melanocytes: Melan-a) and malignant (melanoma SKMEL28 and breast cancer MDA-MB231) cell lines and tested the cells in various relevant assays. As shown in Figure 3a-e, increasing Kindlin-3 expression in SKMEL28 and MDA-MB231 cells significantly increased cell adhesion to collagen I, III and fibronectin and, consistently, induced a decrease in their migratory and invasive activities. In contrast, knockdown of Kindlin-3 in Melan-a cells decreased cell adhesion and increased migration and invasion, thus bringing their phenotype closer to that of the tumor cells (Figure 4A a). Similar to the effect of Kindlin-3 downregulation in Melan-a cells, further knockdown of Kindlin-3 in SKMEL28 and MDA-MB231 cells also decreased cell adhesion and increased migration and invasion (Figure 4A b-e). This was also illustrated by live cell imaging (supplementary movies 1-4), which demonstrated a more disorganized stress-fiber structures and exhibiting abundant filopodia (arrows; actin in red, Figure 4B and movies 1&2) in the direction of migration compared to control cells which appeared to be more adherent. Interestingly, knockdown of Kindlin-3 in SKMEL28 and MDA-MB231 cells also enhanced their proliferation rate and their ability to exhibit anchorage independent growth (Figure 4C). Similar results were obtained with A549 lung cancer and M10 melanoma cell lines. Noteworthy, transcript quantification confirmed that the knockdown of Kindlin-3 was specific as the expression of Kindlin-1 and Kindlin-2 was not affected (Figure 4C c).

**Figure 5:**
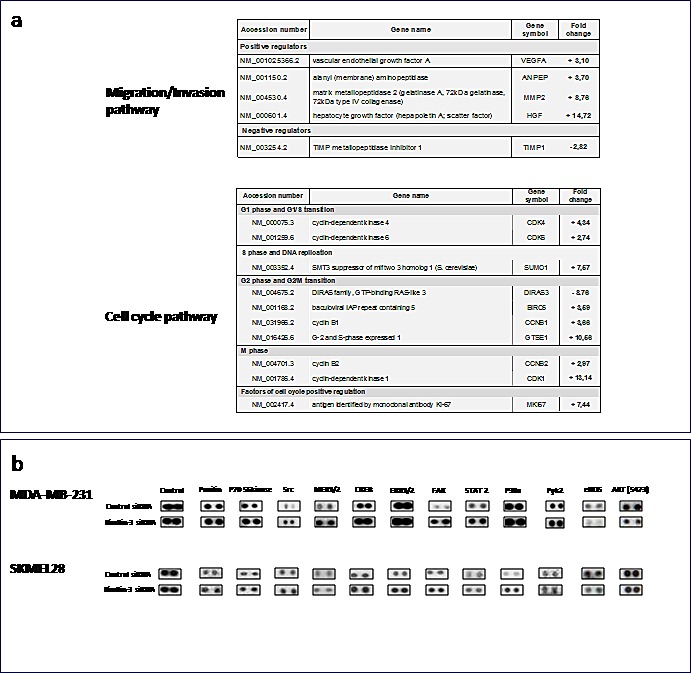
Kindlin-3 mediated signalling pathways in tumor cells a qRT-PCR arrays analyses of Kindlin-3 silenced cells. Total RNA from kindlin-3 silenced tumor cells were analyzed by SignArrays specific for migration/invasion and cell cycle pathways. Variations, means of three independent experiments carried out in duplicate. b. Phosphoproteomic array membranes were probed with proteins from total cell lysates of Kindlin-3 siRNA and control siRNA transfected melanoma cells and signal was detected with chemiluminescence. Representative arrays of three independent experiments are shown.

Gene expression analyses of a set of genes involved in cell cycle, adhesion, migration and invasion, in SKMEL28 and MDA-MB231 cells with downregulated Kindlin-3 expression, demonstrated altered levels of MMP-2, TIMP-1, CDK4, CDK6, and Ki-67 mRNAs, as shown in the table of Figure 5a. In addition, human kinase phosphorylation profile showed increased phosphorylation of FAK, paxillin, and PYK2 (Figure 5b). Taken together, these data suggest that downregulation of Kindlin-3 expression findings is associated with expression of a more malignant phenotype.

### Kindlin-3 mediates talin-dependent β3 integrin activation in tumor cells

**Figure 6:**
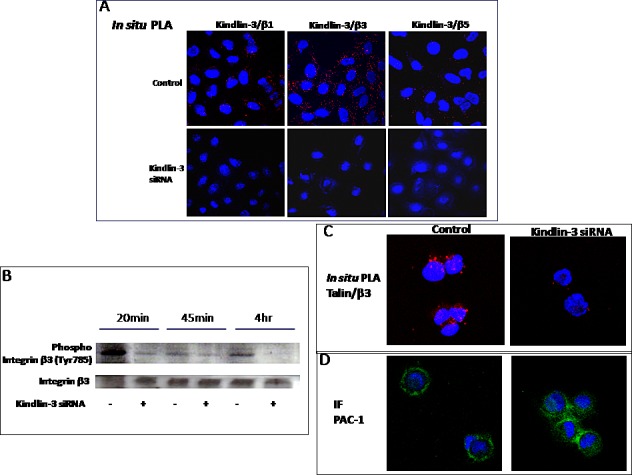
Role of Kindlin-3 in integrin function in tumor cells A Kindlin-3 interacts with integrin beta chains in tumor cells. *In situ* Proximal ligation assay (*In situ* PLA) using confocal microscopy shows red fluorescent spots which denotes very close localization between Kindlin-3 and integrins β1, β3 and β5. Fluorescence was markedly decreased when Kindlin-3 was silenced by siRNA. Nuclei were stained with DAPI (blue). B. Kindlin-3 silencing decreases β3 integrin phosphorylation. The phosphorylated Tyr785 of β3 integrin was analyzed by Western blot in melanoma cells SKMEL28 silenced by siRNA transfection for Kindlin*-3* expression. Representative images and blots of three independent experiments are shown. C. Kindlin-3 is required for Talin / β3 integrin interaction in tumor cells. *In situ* PLA shows a markedly decreased fluorescence, reflecting decreased Talin/ β3 integrin interaction, when Kindlin-3 was silenced by siRNA in SKMEL28 cells. Nuclei were stained with DAPI (blue). D. Detection of an intracellular PAC-l epitope in SKMEL28 cells. Cells were allowed to adhere for 45 min and were then, without fixation, labeled for staining of anti-human mAb PAC-1: FITC IgM (20μg/ml) followed by fixation with 0.5% paraformabdehydel. Cells were visualized in confocal microscope.

Because Kindlins are known to bind integrins, we asked whether Kindlin-3 binds integrins in the malignant cell lines, using an in situ proximity ligation assay (PLA), which detects protein-protein interactions. As shown in Fig. 6A, Kindlin-3 was found to interact with integrins β3, β1, and β5 in SKMEL28 and MDA-MB231 cells, as illustrated by the appearance of fluorescent spots, which were abolished after transfection of the cells with Kindlin-3 siRNA. Moreover, Kindlin-3 siRNA administration to SKMEL28 and MDA-MB231 cells reduced the formation of talin-integrin complexes (Figure 6C) necessary for integrin activation [28]. Consistently, the activation status of integrin β3 was reduced, as determined by an antibody recognizing phosphorylated tyrosine 785 (Figure 6B). This tyrosine is located in the membrane distal tail region NxxY of integrin 1, 2 and 3 to which Kindlin-3 directly binds [29].

Furthermore, adhesion assays where cells were allowed to adhere for 45min and then examined using confocal microscopy after 1hr incubation with PAC-1:FITC antibody, demonstrate that the strong membrane localization of PAC-1 antibody in control tumor cells. This antibody which binds specifically to the activated form of β3-integrin, appeared intracellularly in the Kindlin-3 knockdown cells (Figure 6B-D) [30, 31]. PAC-1, which was mainly studied in platelets, has also been used to study the active form of β3 in tumor cells such as melanoma [32, 33]. Thus, these data suggest that Kindlin-3 mediates talin-dependent integrin activation, at least in part, through the regulation of integrin activation and internalization, with consequences on cell attachment and migration [34, 35].

### Kindlin-3 knockdown promotes metastasis formation through Integrin mediated effects

**Figure 7:**
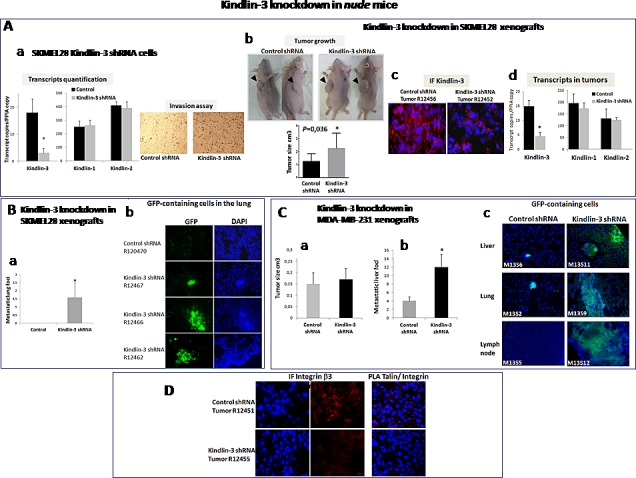
Kindlin-3 knockdown promotes metastasis formation through Integrin mediated effects A **Stable Kindlin-3 knockdown enhances** *in vitro* **invasion and** *in vivo* **tumor growth** a. Transcript quantification by qRT-PCR of Kindlin-1, Kindlin-2 and Kindlin-3 in melanoma SKMEL28 cells stably transfected with Kindlin-3 shRNA (top); Columns, means of three independent experiments carried out in triplicate; bars refer to 95% confidence intervals; *, *P* <0.05. Cell invasion on matrigel coated filters using a modified Boyden chamber (bottom). Stably transfected SKMEL28 cells were plated at a density of 2.0 × 10^4^ per insert. Medium with 10% FBS was in the lower chamber as a chemo-attractant. After 24h of incubation, invasive cells on the lower surface were counted after fixing and staining. Shown are representative data of three independent experiments. b. Subcutaneous xenograft mouse model with Kindlin-3 inactivation. Stable Kindlin-3 silencing by shRNA in SKMEL28 cells enhanced tumor growth. Representative photos of tumors 5 weeks after injection. Results represent two independent experiments with 5 mice in each group. Columns, means of tumor volume; bars refer to 95% confidence intervals; *, *P*=0.036. c. Immunohistochemical (IF) analysis of Kindlin-3 in xenograft tumors from control or Kindlin-3 shRNA SKMEL28 cells 5 weeks after injection, confirms Kindlin-3 silencing in cells with Kindlin-3 shRNA. Representative IF images of 20 primary tumors analyzed are shown. d. Transcript quantification of Kindlin1, Kindlin-2 and Kindlin-3 in the xenograft tumors by qRT-PCR using human specific primer and probe sets. Columns, means of 20 primary tumors analyzed; bars refer to 95% confidence intervals; *, *P*<0.05. **B. Kindlin-3 knockdown promotes lung metastasis formation in SKMEL28 melanoma model** a. Number of metastatic lung foci in Kindlin-3 shRNA-GFP and Control shRNA-GFP injected mice. Columns, means of 20 analyzed lungs; bars refer to 95% CI; *, *P*<0.05. b. Fluorescence analyses of lung tissue sections from GFP-control shRNA and GFP-Kindlin-3 shRNA inoculated SKMEL28 tumor cells showing GFP-positive micrometastases in mice bearing Kindlin-3-shRNA. Representative IF images of 20 lung tissues analyzed are shown. **C. Kindlin-3 knockdown promotes metastasis formation in MDA-MB-231 breast cancer model** a. Tumor growth analysis of Kindlin-3 shRNA vs control xenografts. Results represent two independent experiments with 5 mice in each group. Columns, means of tumor volume; bars refer to 95% confidence intervals. b. Number of metastatic liver foci in Kindlin-3 shRNA-GFP and Control shRNA-GFP injected mice. Columns, means of 20 livers analyzed; bars refer to 95% confidence intervals; *, *P*<0.05. c. Fluorescence analyses of liver, lung and lymph node tissue sections from GFP-control shRNA and GFP-Kindlin-3 shRNA inoculated MDA-MB-231 tumor cells showing larger GFP-positive micrometastases in mice bearing Kindlin-3-shRNA. Representative IF images are shown from 20 mice. D. Kindlin-3 silencing reduced Integrin-β3 phosphorylation (p-Tyr785) and talin-Integrin interaction in vivo shown by Immunofluorescence analysis (left) and *In situ* PLA (right). Nuclei were stained with DAPI (blue). Representative images of 10 primary tumors analyzed are shown.

To investigate the role of Kindlin-3 in tumor growth and metastasis, we generated SKMEL28 and MDA-MB-231 cells with stable knockdown of *Kindlin-3* (shRNA-GFP). Reduction in Kindlin-3 expression did not affect the expression of either Kindlin-1 or Kindlin-2 and was associated with an increased invasive potential of the cells (Figure 7A a). Control and Kindlin-3 shRNA-expressing cells were subcutaneously inoculated into nude/c mice and examined, five weeks later, for tumor growth and development of spontaneous metastases. SKMEL28 xenografts analysis showed that downregulation of Kindlin-3, significantly increased tumor growth (2.27 ± 0.39 vs 1.27± 0.19 cm^3^; *P*=0.036), (Figure 7A b). The decrease in Kindlin-3 protein expression within the subcutaneous tumors was confirmed by immunohistochemical analyses of the tumor sections (Figure 7A c) and by transcript quantification, which also show that Kindlin-3shRNA did not alter the expression of Kindlin-1 and Kindlin-2 in the tumor (Figure 7A d). Histological analyses of the lung tissues also revealed that reduced levels of Kindlin-3 expression correlated with enhanced formation of lung metastatic foci in 7/10 mice inoculated with Kindlin-3 shRNA-expressing SKMEL28 cells. In contrast, no metastases were detected in lungs of mice receiving control SKMEL28 cells (Fig. 7B). In order to verify whether these in vivo results are not limited to the SKMEL28 cells, the effect of Kindlin-3 silencing on metastasis was also investigated on the breast tumor model MBA-MB-231. Despite the lack of significant effect of Kindlin-3 knockdown on tumor growth using these MBA-MB-231 cells, there was a clear increase in metastasis formation in the liver, lung and lymph nodes (Figure 7C). It is interesting however that although the sizes of the metastatic foci were much greater in the shRNA-Kindlin-3 mice (Figure 7C immunofluoresence), the increased number of foci was only statistically different in the liver and lymph nodes (*P*<0.05 for both).

In agreement with the *in vitro* data, Kindlin-3 silencing *in vivo* also decreased integrin β3 phosphorylation at Tyr785 and consistently disrupted talin/integrin β3 interaction in SKMEL28 tumors (Figure 7D). Collectively, these data demonstrates a suppressive role of Kindlin-3 in tumor growth and metastasis that is mediated in part by its ability to regulate integrin β3 activation.

## DISCUSSION

The results of the present study are consistent with a tumor suppressive role for the *Kindlin-3* gene, which we have shown here to be downregulated in several tumor types namely melanoma, breast and lung cancers, through hypermethylation and deletions. Furthermore, our experimental data in mice suggest that downregulation of Kindlin-3 enhances metastasis formation. Indeed, in SKMEL28, cells known to be poorly metastatic, most of the mice with xenografted with shRNA Kindlin-3 cells displayed lung metastases. When xenografted with shRNA Kindlin-3 MDA-MB-231 cells (known to metastasize in nude mice), the increase in metastasis formation was observed in several organs including liver, lymph nodes and lung. However, in this model we did not observe a significant effect on tumor growth at the time of sacrifice. These in vivo studies illustrate that Kindlin-3 plays a more important role in the metastatic process. Based on our *in vitro* data, the anti-metastatic effect of Kindlin-3 is likely to be derived from its ability to regulate cell adhesion, migration and invasion, which are known to be cellular activities that play key roles in the dissemination of tumor cells.

Our mechanistic *in vitro* and *in vivo* data suggest that the changes in the adhesive and migratory properties of tumor cells result from Kindlin-3-mediated regulation of integrin β3 function. This is based on the demonstration that upon Kindlin-3 silencing, both the phosphorylation and interactions with talin of integrin β3 were significantly reduced. Indeed, the specific binding to the cytoplasmic tail of integrin's β subunit by the intracellular protein talin has been suggested to be a key step of inside-out signaling [28, 36]. Consistently, these effects were associated with enhanced internalization of the integrin β3 active form, a process known to result in reduced cell attachment and enhanced migration, two cellular activities that contribute to the metastatic phenotype [34, 35]. These findings are compatible with a model by which Kindlin-3 suppresses malignant properties of cancer cells by interfering with integrin function. This action of Kindlin-3 is similar to that displayed by PTEN, a known tumor suppressor gene, which has also been shown to antagonize interactions of integrins with the extracellular matrix and integrin-triggered signaling pathways by dephosphorylating focal adhesion kinase (FAK), resulting in inhibition of integrin-mediated cell spreading, migration, and invasion [37].

The two other members of the Kindlin family, Kindlin 1 and Kindlin-2 have already been reported to be deregulated in solid tumors, more often upregulated. Kindlin-1, but not Kindlin-2 or Kindlin-3 was upregulated in colorectal and lung tumors [38]. Kindlin-1 levels were also associated with increased lung metastasis in breast cancer [20]. Kindlin-2 was overexpressed in gastric cancer and was correlated with poor prognosis [39]. It was also increased in malignant mesothelioma [40]. However, a suppressive role of Kindlin-2 in mesenchymal cancer cell invasion was also reported as high levels of Kindlin-2 were associated with a decrease in the invasion potential [41]. The functional consequences of the down regulation of Kindlin-3 in tumor cells demonstrated in our study also suggest that Kindlin-2 and Kindlin-3 play a different and therefore non-redundant role in cancer.

The mechanism of how Kindlin-2 and Kindlin-3 play opposing roles in tumor suppression is not clear since the literature suggests that both interact with similar residues of beta integrin subunits. However, Bialkowska *et al*. [11] have shown nevertheless that the recognition of integrin by these 2 Kindlins, -2 and -3 is not identical. These may suggest that the different kindlins may play different function in cancer. Kindlin-2 is expressed at higher levels than Kindlin-3 in most cells and it is possible that a critical concentration of this Kindlin is necessary for optimal function. Indeed, a suppressive role of Kindlin-2 in mesenchymal cancer cell invasion was also reported as high levels of Kindlin-2 were associated with a decrease in the invasion potential [41]. In this context, a very recent report by Plow's group [42], linked Kindlin-3 overexpression in breast tumor cells to increased metastasis. These studies, together with our present results, highlight the complex role of Kindlin members which could exhibit dual effects. Future investigations will no doubt shed light on the mechanisms involved.

*Kindlin-3* is known as a LAD-III causing gene. Its somatic alterations in cancer suggest that physiological outcomes depend on cellular context. This was also described for the tumor suppressor genes *PTEN* and *ATM*, whose inactivation also leads to neuronal loss when the mutations are in the germline [43, 44]. Kindlin-3 germline mutations have not yet been shown to predispose LAD-III patients to cancer. It is tempting to speculate that any cancer promoting advantage brought by Kindlin-3 germline inactivation may be counterbalanced by the associated defects in tumor promoting thrombosis [45] and inflammation [46] caused by these mutations.

In summary, this study is, as far as we know, the first demonstration of a putative tumor suppressor role of Kindlin-3 in solid human tumors that is mediated in part by dysregulation of integrin function. These findings suggest that Kindlin-3 represents a novel target that may improve the anti-tumor effects of anti-integrin therapies.

## MATERIALS AND METHODS

### Patients and cell lines

### Patients

Tumor and normal tissue samples were collected from patients with breast cancer (tumor n=129; normal n=23), lung cancer (tumor and normal matched pairs n=18), kidney cancer (tumor n=15; normal n=11) or melanoma (tumor n=71; normal skin n=17 and nevi n=11). Written informed consent was obtained from each patient according to the recommendations of the local ethics committee. The study was approved by the INSERM review board and ethic committee (IRB, number X80). PMN cells from healthy donors were isolated by Ficoll centrifugation and cryopreserved using standard procedures. PMN and tumoral gDNA were extracted with the DNA blood mini kit and DNA Tissue Kit (Qiagen) respectively.

### Cell lines

Human breast cancer cells MDA-MB-231 and T47D, human melanoma cells SKMEL28 and human lung cancer cells A549, were obtained from the American Type Culture Collection (ATCC Manassas, VA). Human melanoma cells M10 established from the primary nodular melanoma of a patient, were cultured as previously described [47]. Melan-a is a nontransformed mouse melanocyte line that retains many of the characteristics of normal melanocytes [48].

### Expression Analyses

### Real-Time quantitative PCR (qRT-PCR)

Total RNA was extracted from frozen tissue sections or cells using TRIzol reagent (Invitrogen, San Diego). RNA quantity and quality was assessed using the Nanodrop-ND-1000 (Nanodrop Technologies, Wilmington). First-strand cDNA was synthesized using a High-Capacity cDNA Archive Kit (Applied-Biosystems) according to the manufacturer's protocol. *Kindlin-1*, *Kindlin-2* and *Kindlin-3* primer and probe sets (sequences are listed in supplementary Materials, available online) were specifically designed for each transcript and span 2 exons in order to avoid DNA contaminations and pseudogenes (Eurogentec, Belgium). Transcript levels were measured by qRT-PCR using Perfect Master Mix-Probe (AnyGenes, France) on LightCycler-480 (Roche) according to the manufacturer's protocol. The transcript levels were normalized to the housekeeping *PPIA* (peptidylprolyl isomerase A) transcripts.

### Immunohistochemistry

Tumor sections were analyzed by immunohistochemical staining using 2 different antibodies directed against Kindlin-3, Abcam ab68040 [49] and anti-Kindlin-3 antibody produced against a human kindlin-3 specific peptide corresponding to amino acids 19 –31 (RVFVGEEDPEAES) and affinity purified on a sepharose matrix; Genosphere France (Figure 1C) [50]. Both antibodies are specific to Kindlin-3 and do not recognize either Kindlin-1 or Kindlin-2 (Fig 1C). Staining was visualized using the Vectastain Elite universal ABC-kit (Vector-Laboratories, Burlingame), followed by counterstaining with Mayer's hematoxylin. Three-amino-9-ethyl-carbazole (Sigma-Aldrich, France) was used as the chromogen.

### Western Blotting analyses

Western Blots were performed as described previously [51] using anti-Kindlin-3 antibody (Genosphere) or anti-phospho-Integrin β3 (pTyr785; Sigma) normalized to actin or β3-integrin respectively.

### Human Phospho-Kinase Array

The human phospho-Kinase Array Kit (Proteome Profiler Array, ARY003, R&D Systems, UK) was used to detect relative levels of phosphorylation of 46 kinase phosphorylation sites, according to the manufacturer's instructions, using total cell lysates of cells transfected with either Kindlin-3 or scrambled siRNA. Total cell lysates were diluted to 150 to 200 μg/mL protein in a detergent-, urea-, and phosphatase inhibitor-containing solubilizing buffer (R&D Systems) and incubated with the arrays overnight at 4°C. After washing away the unbound material, membranes were incubated with a cocktail of phosphosite–specific, biotinylated antibodies, to detect phosphorylated kinases with streptavidin-horseradish peroxidase and signals were revealed with a chemiluminescent substrate kit (ECL Dura Thermo Scientific, 34076). Independent experiments were performed in duplicate.

### Pathway Specific Gene Expression Profiling

Signalling pathways transcript analyses were conducted using Human qPCR SignArrays^®^ 96 system (cell cycle, adhesion, migration and invasion gene profiling analysis Human qPCR SignArrays® 96 kit; and Perfect MasterMix SYBR Green^®^ (AnyGenes, France) on a LightCycler 480 (Roche, France) as described by the manufacturer, in SKMEL28 and MDA-MB231 cells with downregulated Kindlin-3 by siRNA, 24h after transfection. Quality control of qPCR data for consequent analysis was based on control for genomic DNA contamination and positive and negative PCR controls. Briefly, a total volume of 20μl of PCR mix, which included 10μl of MasterMix, 8μl of double distilled water and 2μl of cDNA was loaded into each of the 96 wells of the qPCR array. PCR amplification was conducted at 95°C for 10 min, followed by 40 cycles of 95°C for 10 sec and 60°C for 30 sec. The mRNA expression for each gene was normalized using the average expression of 8 housekeeping genes: *peptidylprolyl isomerase A (cyclophilin A, PPIA), b-actin (ACTB), TATA box binding protein (TBP), beta-2-microglobulin (B2M), ribosomal protein large PO (RPLPO), hypoxanthine phosphoribosyltransferase 1 (HPRT1), transferring receptor (p90, CD71) (TFRC)* and *glucuronidase beta (GUSB)*. Gene profiling analyses were performed in three independent experiments. The ΔΔCt method was used for data analysis. Data analysis was conducted using Excel spreadsheets in Windows. Fold changes were calculated for each gene as the difference in gene expression between cells transfected with *Kindlin-3* siRNA and control siRNA using the CS2R software provided by the manufacturer.

### Genetic Analyses

### Genome-wide DNA array analysis

Genomic DNAs from melanoma tissues (n=15) were analyzed by high-density array comparative genomic hybridization (CGH) technologies using the 1 × 1M Microarray SurePrint G3 (Agilent Technologies) according to the manufacturers' recommendations. Analyses were performed using the Genomic Workbench software (Agilent Technologies) with the help of the ADM-2 algorithm for array CGH data, Genomic Suite 6.5 software (Partek). The final retained abnormalities were validated by two investigators by visual inspection considering the size and Log2 ratios of the abnormalities with respect to the individual background noise of each array at each particular chromosomal location, as reported previously [52]. Polymorphic copy number variations were excluded using the Database of the Genomic Variants tracks in the University of California Santa Cruz Genome Browser or in the Genomic Workbench software.

### Copy number quantification

*Kindlin-3* relative copy number was determined by qPCR (cycle conditions available upon request) using the LightCycler-480 System. Total DNA content was estimated by assaying *GAPDH* for each sample, and 20 ng of gDNA was mixed with the SYBR-Green-I Master (Roche) and 2 pmol/μl of each primer.

### Oncomine database queries

(https://www.oncomine.org) were performed using the same criteria for all tested cancer types. Briefly, we searched all gene expression datasets for *Kindlin-3* in the “cancer versus normal analysis” category.

### Bisulfite conversion and pyrosequencing

Quantitative DNA methylation analysis was performed by pyrosequencing of bisulfite treated DNA [53]. One μg of DNA was bisulphite converted using the EpiTect 96 Bisulfite kit (Qiagen) according to the manufacturer's instructions. Regions of interest were amplified using 30 ng of bisulfite treated human genomic DNA and 5 to 7.5 pmol of forward and reverse primer, one of them being biotinylated. Sequences for oligonucleotides for PCR amplification and pyrosequencing are given in supplementary Materials (available online). Reaction conditions were 1x HotStar Taq buffer supplemented with 1.6 mM MgCl2, 100 μM dNTPs and 2.0 U HotStar Taq polymerase (Qiagen) in a 25 μl volume. The PCR program consisted of a denaturing step of 15 min at 95°C followed by 50 cycles of 30 s at 95°C, 30 s at the respective annealing temperature and 20 s at 72°C, with a final extension of 5 min at 72°C. Ten μl of PCR product were rendered single-stranded as previously described [53] and 4 pmol of the respective sequencing primer were used for analysis. Quantitative DNA methylation analysis was carried out on a PSQ 96MD system with the PyroGold SQA Reagent Kit (Qiagen) and results were analyzed using the Q-CpG software (V.1.0.9, Biotage AB).

### 5-Aza-2′ deoxycytidine treatment

Tumor cell lines were treated with demethylating agent 5-Aza-2′deoxycytidine (Sigma-Aldrich) for 5 days at a concentration of 5μM. Total RNA extracted from treated and untreated cell lines were reverse transcribed analyzed by real time quantitative PCR for *Kindlin-3* and *PPIA* gene expression.

### Expression vectors and Small interfering RNA transfections

The complete open reading frame of the human *Kindlin-3* gene was amplified by PCR, cloned into the pcDNA 3.1-GFP TOPO-TA mammalian expression vector (Invitrogen), and transiently transfected into MDA-MB-231, SKMEL28 tumor cells and Melan-a melanocytes. Control vector (pcDNA3.1-GFP) containing no insert was used to generate negative control cells. Vectors were sequenced in the forward and reverse directions to verify the insertion of the *Kindlin-3* and GFP sequences and the lack of insert in the control plasmids.

For Kindlin-3 knockdown, two different Kindlin-3 siRNA (IDs:s38130 and Ids:s38131; Applied-Biosystems) and scrambled siRNA oligos (Control) were used with Lipofectamine-2000 (Invitrogen) as transfectant.

### Cellular assays

### Functional *in vitro* assays

Adhesion assays were performed on cells incubated with immobilized integrin ligands (fibronectin, collagen I and collagen III), in triplicate, for 30 min at 37 °C. After washing, cells were fixed and treated with 0.5% crystal violet staining. An automated plate reader was used to quantitate cell attachment, once each well was solubilized with Sorenson solution, and OD read at 590nm.

The *in vitro* migration (on uncoated filters) and invasion (on coated filters with matrigel) were performed using a modified Boyden chamber [54] in 24-well plates and 8-mm pore filter inserts (BD Bioscience).

Proliferation assay on transfected cells seeded on 96-well plates was measured using the CellTiter 96 Aqueous Non Radioactive Cell Proliferation Assay (Promega).

For anchorage-independent assays, transfected cells were mixed with 1 ml of growth media and 0.3% agar and then layered onto 0.5% agar beds in 60-mm diameter Petri dishes. Cells were allowed to grow for 10 days and the entire area of the dish was counted. Colonies were counted and the plates were photographed. Assays were conducted in triplicate.

### Immunofluorescence microscopy and *In situ* proximity ligation assay (*In situ* PLA)

Cells were fixed and incubated with anti-Kindlin-3 antibody (Genosphere), followed by Alexa 488 secondary antibody and examined with a laser-scanning confocal microscope (Leica-Lasertechnik, Heidelberg). In the negative controls the primary antibody was substituted with PBS.

*In situ* PLA used to assess protein-protein interactions, cells grown on Lab-tek chamber slides (Nunc, #154534), transfected with *Kindlin-3* or control siRNA and immediately ﬁxed were subjected to In situ PLA using the Duolink Detection kit (Olink Bioscience, Sweden) according to the manufacturer's instructions. Brieﬂy, slides were blocked, incubated with antibodies directed against Kindlin-3 (Genosphere), Integrins β1, β3 or β5 (Abcam ab58524, Santa Cruz sc-14009, sc-14010) or Talin (Abcam ab11188) and thereafter incubated with PLA probes, which are secondary antibodies (anti-rabbit and anti-mouse) conjugated to unique oligonucleotides. Circularization and ligation of the oligonucleotides was followed by an ampliﬁcation step. The products were detected by a complementary ﬂuorescently labeled probe. Protein complexes were visualized in a laser-scanning confocal microscope (Leica-Lasertechnik) as bright fluorescent signals.

### PAC-1 Immunofluorescence

Active conformation of αIIbβ3 integrin in melanoma cells was essentially performed as already described [31]. Briefly, tumor cells plated on coverslips and allowed to adhere for 45 min were incubated for 1 hr with the mouse anti-human mAb PAC-1:FITC IgM (20μg/ml) that specifically recognizes the high-affinity αIIbβ3 integrin [30] or a mouse IgM:FITC used as control antibody (Becton Dickinson, France). Adherent cells were then fixed with 0.5% paraformaldehyde prior to visualization by confocal microscope.

### Live imaging of cells

Live fluorescent microscopy of adhesion/migration dynamics was performed 30min after plating SKMEL28 cells expressing RFP-actin on IBIDI plates (IBIDI HI-q4). Imaging was performed using 20x and 40x objectives on a Nikon BioStation IM Live Cell Recorder (Nikon Instruments Inc.) and image acquisition was done every 3 min. For actin labelling, SKMEL28 cells were transduced with RFP-actin using BacMam reagent (Invitrogen, CellLight® Reagent BacMam 2.0). The reagent was directly added to the cells at approximately 60% confluence followed by incubation for 16 hr at 37°C, after which the cells were trypsinized, plated and imaged as above.

### Tumor formation in a xenograft model

Mouse experiments were conducted according to French veterinary guidelines and those formulated by the council of Europe for experimental animal use (L358-86/609EEC). SKMEL28 and MDA-MB-231 cells were stably transfected with psiRNA-h7SK vector with small hairpin RNA (shRNA) targeting *Kindlin-3* or with shRNA control (Invivogen, France). Female 6-week-old nude/c mice were injected subcutaneously with 5×10^6^ SKMEL28 and 3×10^6^ MDA-MB-231 stably transfected *Kindlin-3* shRNA or control shRNA cells (n=10 control-GFP and n=10 Kindlin-3-shRNA-GFP cells, for each cell line). Tumor size was measured twice a week by caliper. Five weeks later, all mice were sacrificed by cervical dislocation, and primary tumors were removed and measured with calipers. Tumor volumes were calculated as: volume (cm^3^)=*a*×*b*^2^/2 (*a* and *b* are the two registered perpendicular diameters, with *a* > *b*). For metastasis analyses, lungs for SKMEL28 and lungs, livers and lymph nodes for MDA-MB-231, were also dissected and metastatic foci were searched at different levels on serial sections (every 300 μm) in each organ and analyzed using Axiovert fluorescent microscopy. The number of metastatic foci was determined in a blinded manner by two independent pathologists.

### Statistical analyses

The Student *t* test was used to compare continuous variables between two groups. Data were considered statistically significantly different for *P* values of .050 or less. Pairwise associations between the different groups were assessed by the use of Spearman rank correlation coefficient. All statistical tests were two-sided. Analyses were performed using Prism 6 (GraphPad Software Inc, La Jolla, CA).

## SUPPLEMENTARY MATERIAL, FIGURES AND TABLES






