# Supplementary material for: A novel tumor suppressor function of Kindlin-3 in solid cancer
Source: Oncotarget. 2014 Jun 18;5(19):8970–85. doi: 10.18632/oncotarget.2125 (PMC4253411; doi:10.18632/oncotarget.2125)
Supplement: Supplementary file 1 [file oncotarget-05-8970-s001.pdf]

A novel tumor suppressor function of Kindlin-3 in solid cancer

Supplementary Methods

Supplementary Table 1: Real-time qPCR primers

**Gene name**	**Primer sequences**
KINDLIN-1	Forward : 5'-AAGCACTTGCGGATATGTACC-3'
	Reverse : 5'-TCCTCTTGGATGCCTTGTTC-3'
	Probe : FAM-AGGTTGGCTAGACTCCTCACGCTCCCT-TAMRA
KINDLIN-2	Forward : 5'-CTCCTGAATGTTTGGTGTCTCC-3'
	Reverse : 5'-CTCATCTTGGCTTCAATTAGACTC-3'
	Probe : FAM-AGAACAAGCAGATAACAGCGAGAATCTTGGA-TAMRA
KINDLIN-3	Forward : 5'- GGCATCGCCAACAACC -3'
	Reverse : 5'- CACTGGCGCATGTTGC -3'
	Probe : FAM- CGACGTGGTCAAGACCTGGCGT -TAMRA
PPIA	Forward : 5'-GTCAACCCCACCGTGTTCTT-3'
	Reverse : 5'-CTGCTGTCTTTGGGACCTTGT-3'
	Probe : FAM-TGGGCCGCGTCTCCTTTGAGCT-TAMRA

Supplementary Table 2: Bisulfite conversion and pyrosequencing: Sequences for oligonucleotides for PCR amplification and pyrosequencing

Gene	Size bp	Annealingtemp	PCR primer forward	PCR primer reverse	Pyrosequencing primer(s)	CpGs
FERMT3_**AMP1**<br>chr11: 63972739 - 63973057	319	58	GTTTAGGTTGGAGTGTAGTGG	Biotin-CCTAAATTCTCAAATAAAAACC	AGGTTGGAGTGTAGTGG<br>TTGTTTGTATTTTTAGTAGA<br>TTGTTTGTATTTTTAGTAGA<br>TTAAAGTGTTGGGGTTAATA	CpG 1-8<br>CpG 2-8<br>CpG 9-12<br>CpG 13-17
FERMT3_**AMP2**<br>chr11: 63973963 - 63974187	225	58	TTGAGTTTGTATTGAGTTTTTTTT	Biotin-<br>ACAACCCTTACTTATACCTTATCAC	TTTTTAAATGAGGAAGG<br>TTTGTGGGTTGGTAGTG	CpG 1-3<br>CpG 4-7
FERMT3_**AMP3**<br>chr11: 63974517 - 63974797	281	61	GAGTTGTGGGTTGGTATTGAGTA	Biotin-<br>AATAAACAAACCCTAATAAACCTCC	TTGTGGGTTGGTATTGA<br>GTTTGTAGGATGGGAG<br>GTTGGAGAGAGAGTTTGA	CpG 1-4<br>CpG 4-8<br>CpG 9-12

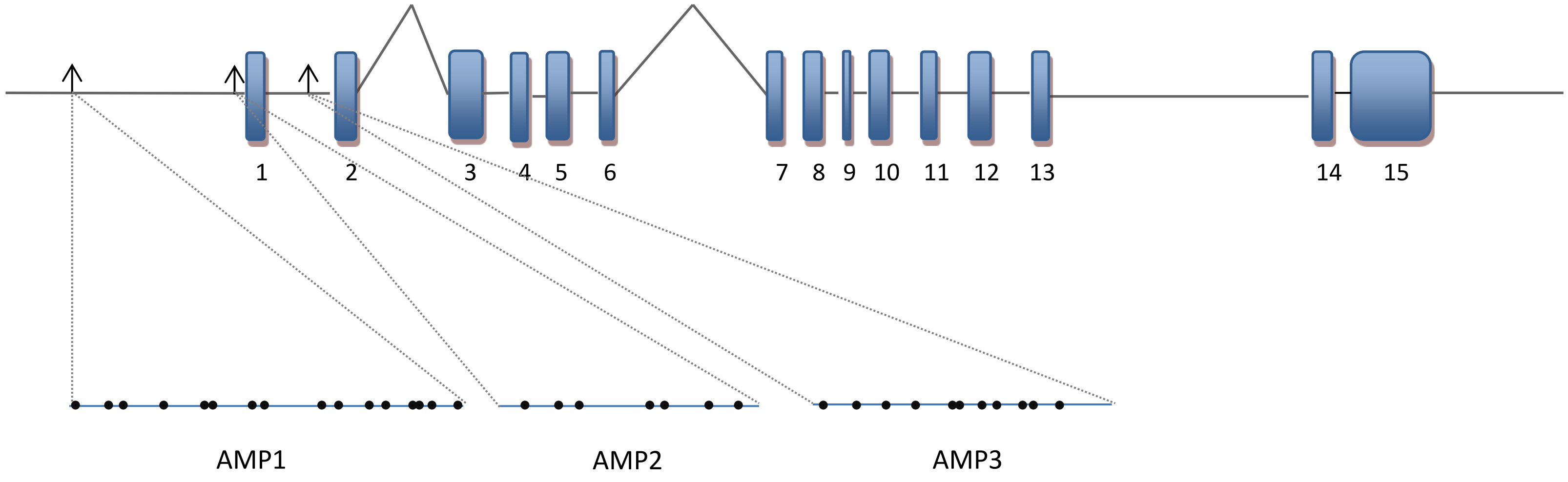

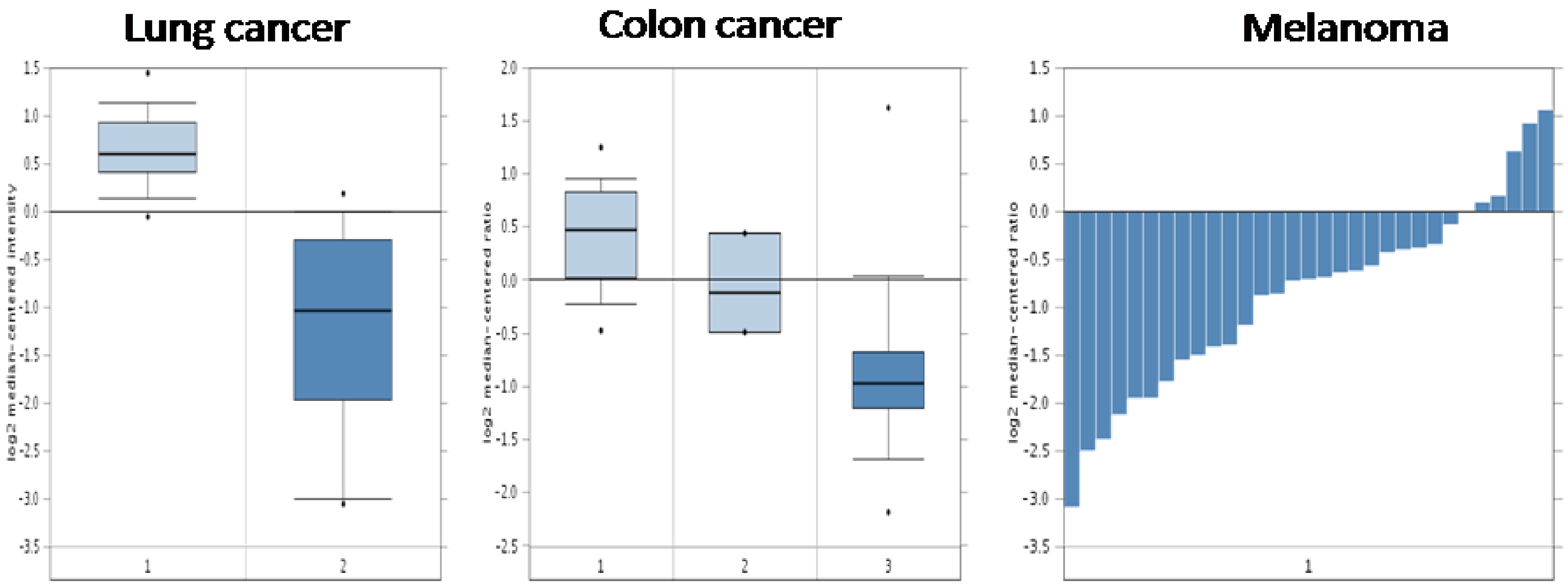

Supplementary Figure 1: *Kindlin-3* expression and regulation in human tumors

a. Analysis of *Kindlin-3* gene expression in solid human tumors using the Oncomine database (lung, tumors (2) n= 91, normal (1) n= 65; colon, tumors (2, rectum and 3, Rectal Adenocarcinoma) n= 63, normal (1) n= 19; Cutaneous Melanoma (1) n=31). Oncomine™ (Compendia Bioscience, Ann Arbor, MI) was used for analysis and visualization. (www.oncomine.com).

a

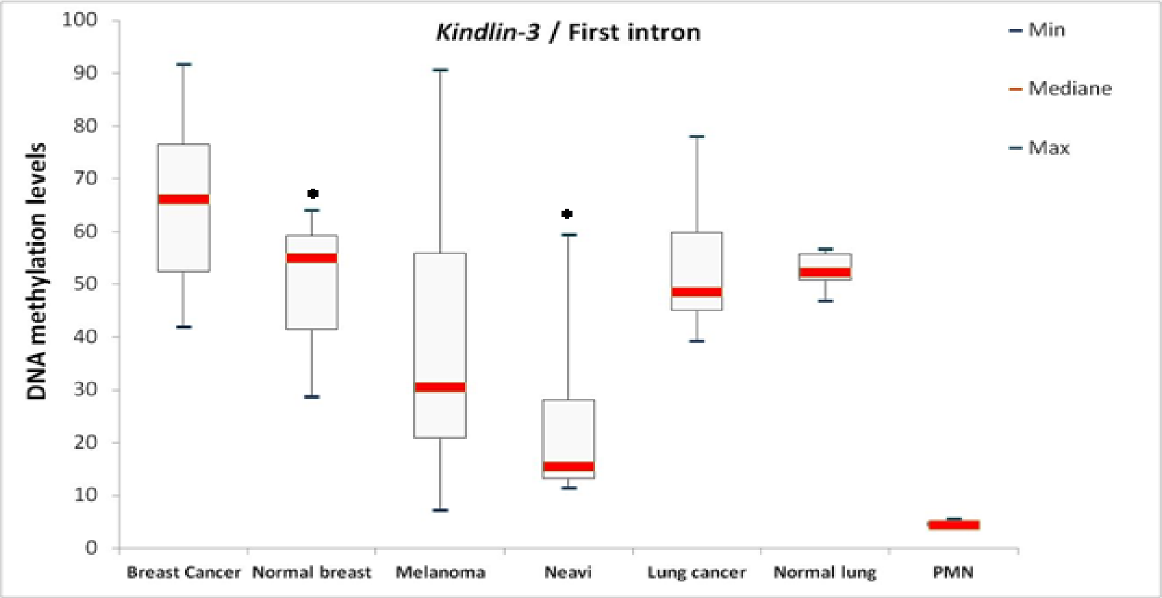
Kindlin-3 / First intron
DNA methylation levels
100
90
80
70
60
50
40
30
20
10
0
Min
Mediane
Max
Breast Cancer
Normal breast
Melanoma
Neavi
Lung cancer
Normal lung
PMN

b

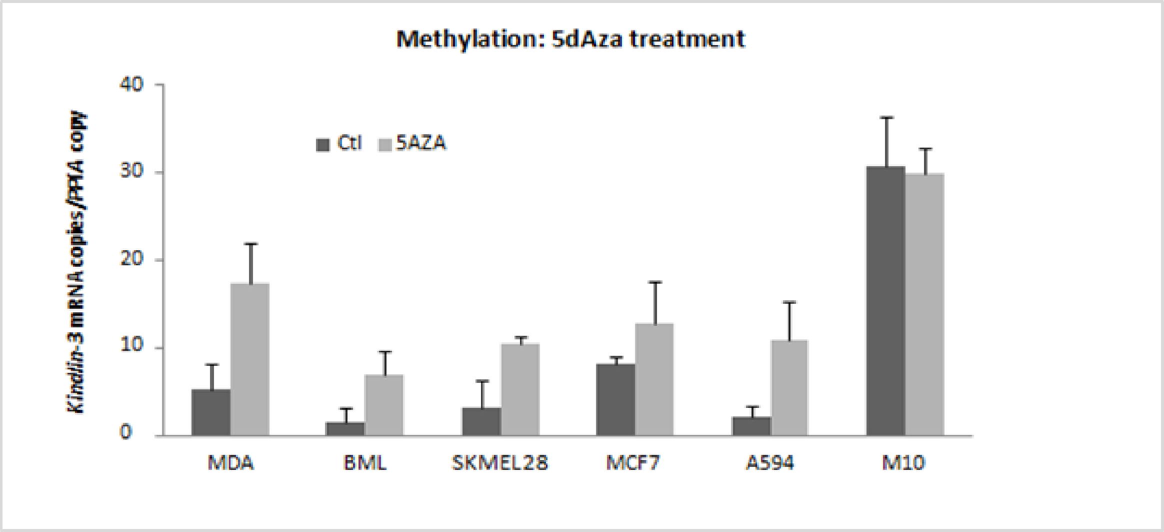
Methylation: 5dAza treatment
Kindlin-3 mRNA copies/PPIA copy
40
30
20
10
0
Ctl
5AZA
MDA
BML
SKMEL28
MCF7
A594
M10

Supplementary Figure 2: *Kindlin-3* regulation in human tumors

a. DNA methylation levels in the CpG island in the first intron of *Kindlin-3* (AMP3) in **human normal and cancer tissues** represented by boxplots. *, *P* <0.05

b. *Kindlin-3* gene expression measured by qRT-PCR in cancer cell lines after treatment with the DNA methylation inhibitor 5-aza-2-deoxycytidine (5-dAza). Columns, means of three independent experiments carried out in triplicate; bars refer to 95% confidence intervals; *, *P*<0.05

Supplementary movies 1 and 2: Kindlin-3 knockdown inhibits cell adhesion and promotes directed cell migration. Frames were taken every 3min for 24 hrs. Note that cells transfected with Kindlin-3 shRNA migrate faster and develop abnormal stress-fiber structures exhibiting abundant filopodia (actin in red) in the direction of migration. By contrast, more control cells display a normal distribution of filamentous actin and appear to be more firmly adherent.

Supplementary movies 3 and 4: Control or Kindlin-3 shRNA SKMEL28 cells were imaged during adhesion. Live cell imaging was performed with Nikon BioStation IM Live Cell Recorder. Frames were taken every 3min for 24 hrs. Note that Kindlin-3 shRNA transfection decreased cell adhesion.
