# Supplementary material for: A novel tumor suppressor function of Kindlin-3 in solid cancer
Source: Oncotarget. 2014 Jun 18;5(19):8970–85. doi: 10.18632/oncotarget.2125 (PMC4253411; doi:10.18632/oncotarget.2125)
Supplement: Supplementary file 2 [file oncotarget-05-8970-s002.pdf]

A novel tumor suppressor function of Kindlin-3 in solid cancer

Supplementary data

Supplementary Table 1

The Cancer genome Atlas (TCGA) analysis of 1123 tumor patient samples. Tumor samples harboring homozygous deletion of the chromosome region 11q13.1 (including *Kindlin-3*) in the TCGA dataset (TCGA; http://cancergenome.nih.gov/; Accessed the 09th June 2012). Data were obtained using Genome-Wide Human SNP Array 6.0 (Affymetrix®) and homozygous deletion was defined by a Log2 Ratio <-0.5.

Tumor type	Homozygously deleted (n)	Homozygously deleted (%)
Breast invasive carcinoma	11/93	12%
Rectum adenocarcinoma	3/50	6%
Lung adenocarcinoma	1/21	5%
Glioblastoma	11/372	3%
Ovarian serous cystadenocarcinoma	17/494	3%
Colon adenocarcinoma	2/137	1%
